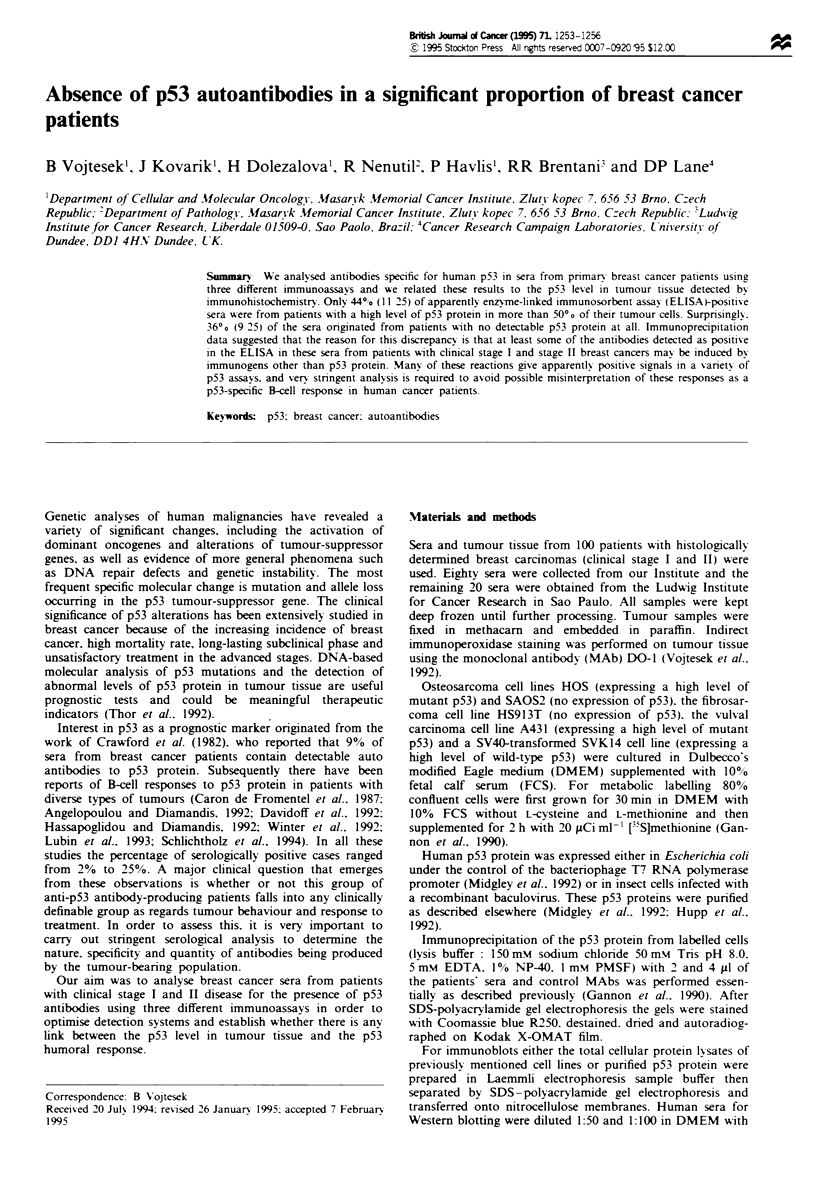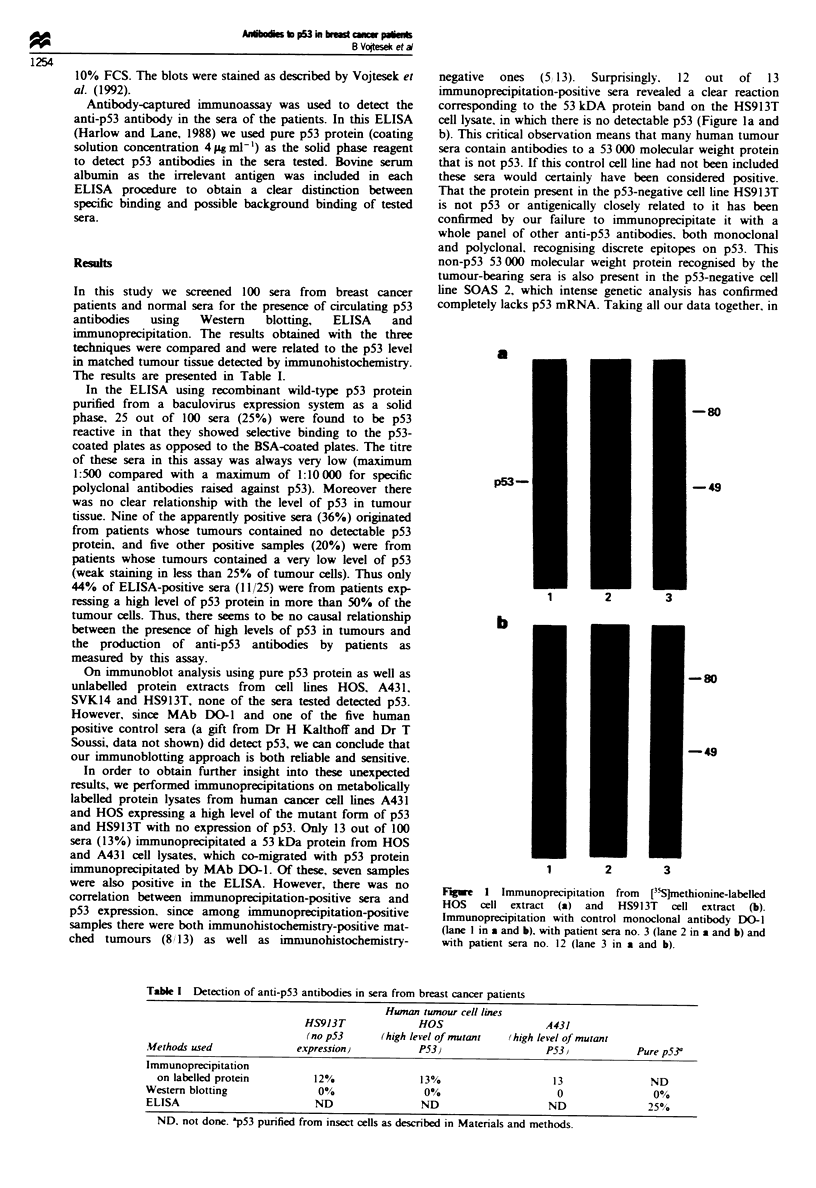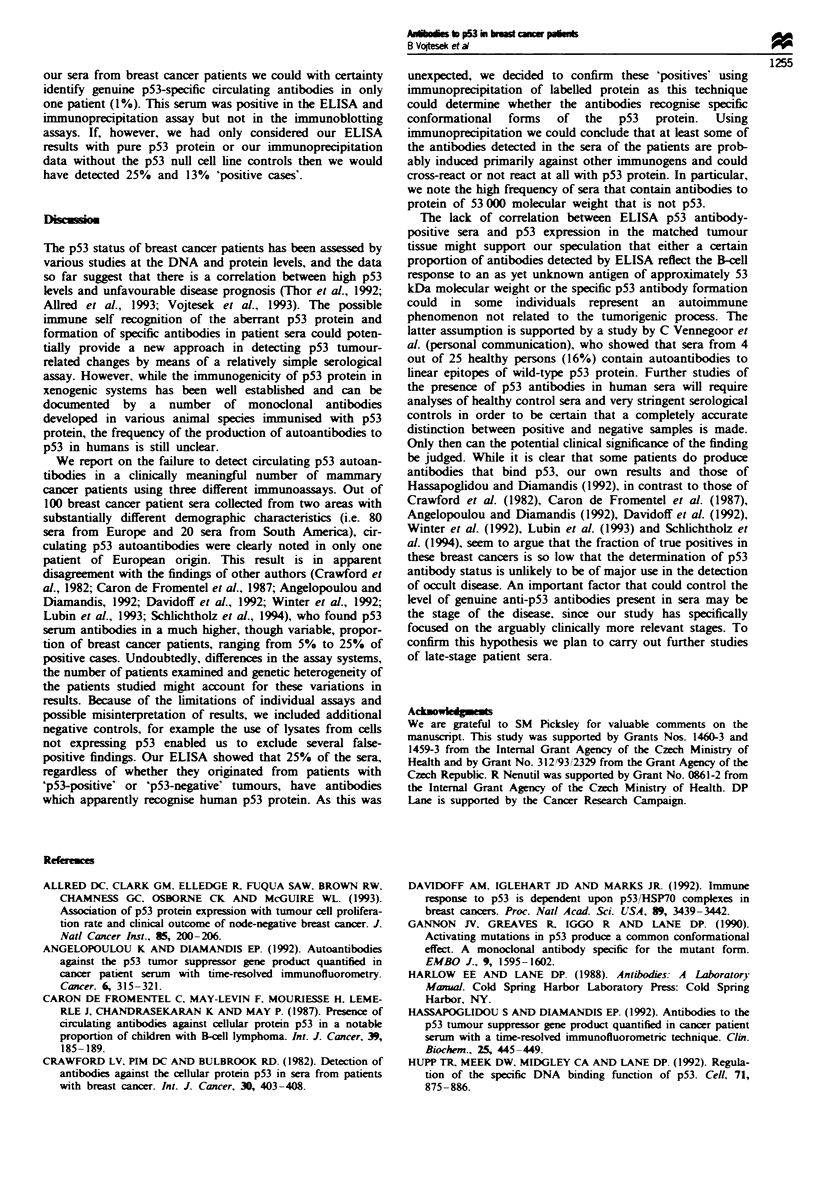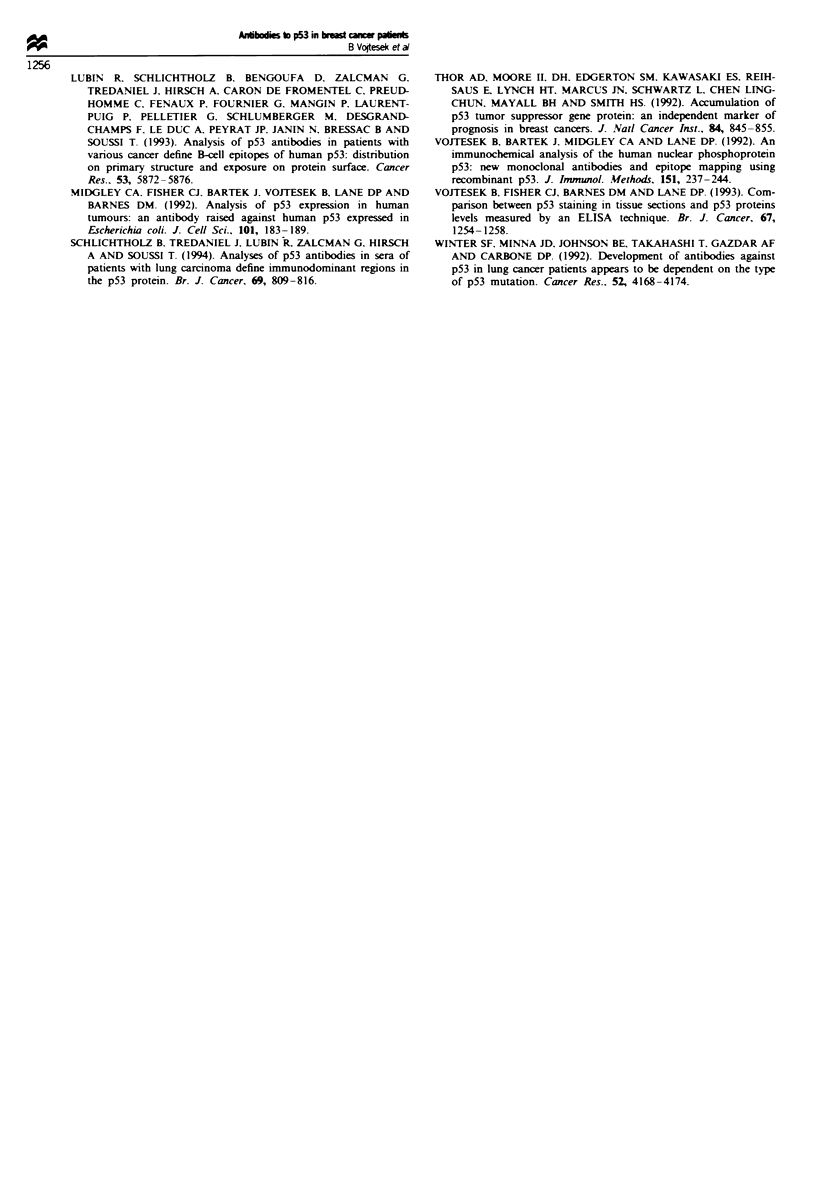# Absence of p53 autoantibodies in a significant proportion of breast cancer patients.

**DOI:** 10.1038/bjc.1995.242

**Published:** 1995-06

**Authors:** B. Vojtesek, J. Kovarik, H. Dolezalova, R. Nenutil, P. Havlis, R. R. Brentani, D. P. Lane

**Affiliations:** Department of Cellular and Molecular Oncology, Masaryk Memorial Cancer Institute, Brno, Czech.

## Abstract

**Images:**


© 1995 Stockton Press All rights reserved 0007-0920/95 $12.00

### Absence of p53 autoantibodies in a significant proportion of breast cancer patients

B Vojtesek[1], J Kovarik[1], H Dolezalova[1], R Nenutil[2], P Havlis[1], RR Brentani[3] and DP Lane[4]

[1]*Department of Cellular and Molecular Oncology, Masaryk Memorial Cancer Institute, Zluty kopec 7, 656 53 Brno, Czech Republic;* [2]*Department of Pathology, Masaryk Memorial Cancer Institute, Zluty kopec 7, 656 53 Brno, Czech Republic;* [3]*Ludwig Institute for Cancer Research, Liberdale 01509-0, Sao Paolo, Brazil;* [4]*Cancer Research Campaign Laboratories, University of Dundee, DD1 4HN Dundee, UK.*

**Summary** We analysed antibodies specific for human p53 in sera from primary breast cancer patients using three different immunoassays and we related these results to the p53 level in tumour tissue detected by immunohistochemistry. Only 44% (11/25) of apparently enzyme-linked immunosorbent assay (ELISA)-positive sera were from patients with a high level of p53 protein in more than 50% of their tumour cells. Surprisingly, 36% (9/25) of the sera originated from patients with no detectable p53 protein at all. Immunoprecipitation data suggested that the reason for this discrepancy is that at least some of the antibodies detected as positive in the ELISA in these sera from patients with clinical stage I and stage II breast cancers may be induced by immunogens other than p53 protein. Many of these reactions give apparently positive signals in a variety of p53 assays, and very stringent analysis is required to avoid possible misinterpretation of these responses as a p53-specific B-cell response in human cancer patients.

**Keywords:** p53; breast cancer; autoantibodies

Genetic analyses of human malignancies have revealed a variety of significant changes, including the activation of dominant oncogenes and alterations of tumour-suppressor genes, as well as evidence of more general phenomena such as DNA repair defects and genetic instability. The most frequent specific molecular change is mutation and allele loss occurring in the p53 tumour-suppressor gene. The clinical significance of p53 alterations has been extensively studied in breast cancer because of the increasing incidence of breast cancer, high mortality rate, long-lasting subclinical phase and unsatisfactory treatment in the advanced stages. DNA-based molecular analysis of p53 mutations and the detection of abnormal levels of p53 protein in tumour tissue are useful prognostic tests and could be meaningful therapeutic indicators (Thor *et al*., 1992).

Interest in p53 as a prognostic marker originated from the work of Crawford *et al*. (1982), who reported that 9% of sera from breast cancer patients contain detectable auto antibodies to p53 protein. Subsequently there have been reports of B-cell responses to p53 protein in patients with diverse types of tumours (Caron de Fromentel *et al*., 1987; Angelopoulou and Diamandis, 1992; Davidoff *et al*., 1992; Hassapoglidou and Diamandis, 1992; Winter *et al*., 1992; Lubin *et al*., 1993; Schlichtholz *et al*., 1994). In all these studies the percentage of serologically positive cases ranged from 2% to 25%. A major clinical question that emerges from these observations is whether or not this group of anti-p53 antibody-producing patients falls into any clinically definable group as regards tumour behaviour and response to treatment. In order to assess this, it is very important to carry out stringent serological analysis to determine the nature, specificity and quantity of antibodies being produced by the tumour-bearing population.

Our aim was to analyse breast cancer sera from patients with clinical stage I and II disease for the presence of p53 antibodies using three different immunoassays in order to optimise detection systems and establish whether there is any link between the p53 level in tumour tissue and the p53 humoral response.

Correspondence: B Vojtesek
Received 20 July 1994; revised 26 January 1995; accepted 7 February 1995

### Materials and methods

Sera and tumour tissue from 100 patients with histologically determined breast carcinomas (clinical stage I and II) were used. Eighty sera were collected from our Institute and the remaining 20 sera were obtained from the Ludwig Institute for Cancer Research in Sao Paulo. All samples were kept deep frozen until further processing. Tumour samples were fixed in methacarn and embedded in paraffin. Indirect immunoperoxidase staining was performed on tumour tissue using the monoclonal antibody (MAb) DO-1 (Vojtesek *et al*., 1992).

Osteosarcoma cell lines HOS (expressing a high level of mutant p53) and SAOS2 (no expression of p53), the fibrosarcoma cell line HS913T (no expression of p53), the vulval carcinoma cell line A431 (expressing a high level of mutant p53) and a SV40-transformed SVK14 cell line (expressing a high level of wild-type p53) were cultured in Dulbecco's modified Eagle medium (DMEM) supplemented with 10% fetal calf serum (FCS). For metabolic labelling 80% confluent cells were first grown for 30 min in DMEM with 10% FCS without L-cysteine and L-methionine and then supplemented for 2 h with 20 $\mu$Ci ml$^{-1}$ [$^{35}$S]methionine (Gannon *et al*., 1990).

Human p53 protein was expressed either in *Escherichia coli* under the control of the bacteriophage T7 RNA polymerase promoter (Midgley *et al*., 1992) or in insect cells infected with a recombinant baculovirus. These p53 proteins were purified as described elsewhere (Midgley *et al*., 1992; Hupp *et al*., 1992).

Immunoprecipitation of the p53 protein from labelled cells (lysis buffer : 150 mM sodium chloride 50 mM Tris pH 8.0, 5 mM EDTA, 1% NP-40, 1 mM PMSF) with 2 and 4 $\mu$l of the patients' sera and control MAbs was performed essentially as described previously (Gannon *et al*., 1990). After SDS-polyacrylamide gel electrophoresis the gels were stained with Coomassie blue R250, destained, dried and autoradiographed on Kodak X-OMAT film.

For immunoblots either the total cellular protein lysates of previously mentioned cell lines or purified p53 protein were prepared in Laemmli electrophoresis sample buffer then separated by SDS-polyacrylamide gel electrophoresis and transferred onto nitrocellulose membranes. Human sera for Western blotting were diluted 1:50 and 1:100 in DMEM with

10% FCS. The blots were stained as described by Vojtesek *et al.* (1992).

Antibody-captured immunoassay was used to detect the anti-p53 antibody in the sera of the patients. In this ELISA (Harlow and Lane, 1988) we used pure p53 protein (coating solution concentration 4 $\mu g\ ml^{-1}$) as the solid phase reagent to detect p53 antibodies in the sera tested. Bovine serum albumin as the irrelevant antigen was included in each ELISA procedure to obtain a clear distinction between specific binding and possible background binding of tested sera.

### Results

In this study we screened 100 sera from breast cancer patients and normal sera for the presence of circulating p53 antibodies using Western blotting, ELISA and immunoprecipitation. The results obtained with the three techniques were compared and were related to the p53 level in matched tumour tissue detected by immunohistochemistry. The results are presented in Table I.

In the ELISA using recombinant wild-type p53 protein purified from a baculovirus expression system as a solid phase, 25 out of 100 sera (25%) were found to be p53 reactive in that they showed selective binding to the p53-coated plates as opposed to the BSA-coated plates. The titre of these sera in this assay was always very low (maximum 1:500 compared with a maximum of 1:10 000 for specific polyclonal antibodies raised against p53). Moreover there was no clear relationship with the level of p53 in tumour tissue. Nine of the apparently positive sera (36%) originated from patients whose tumours contained no detectable p53 protein, and five other positive samples (20%) were from patients whose tumours contained a very low level of p53 (weak staining in less than 25% of tumour cells). Thus only 44% of ELISA-positive sera (11/25) were from patients expressing a high level of p53 protein in more than 50% of the tumour cells. Thus, there seems to be no causal relationship between the presence of high levels of p53 in tumours and the production of anti-p53 antibodies by patients as measured by this assay.

On immunoblot analysis using pure p53 protein as well as unlabelled protein extracts from cell lines HOS, A431, SVK14 and HS913T, none of the sera tested detected p53. However, since MAb DO-1 and one of the five human positive control sera (a gift from Dr H Kalthoff and Dr T Soussi, data not shown) did detect p53, we can conclude that our immunoblotting approach is both reliable and sensitive.

In order to obtain further insight into these unexpected results, we performed immunoprecipitations on metabolically labelled protein lysates from human cancer cell lines A431 and HOS expressing a high level of the mutant form of p53 and HS913T with no expression of p53. Only 13 out of 100 sera (13%) immunoprecipitated a 53 kDa protein from HOS and A431 cell lysates, which co-migrated with p53 protein immunoprecipitated by MAb DO-1. Of these, seven samples were also positive in the ELISA. However, there was no correlation between immunoprecipitation-positive sera and p53 expression, since among immunoprecipitation-positive samples there were both immunohistochemistry-positive matched tumours (8/13) as well as immunohistochemistry-negative ones (5/13). Surprisingly, 12 out of 13 immunoprecipitation-positive sera revealed a clear reaction corresponding to the 53 kDA protein band on the HS913T cell lysate, in which there is no detectable p53 (Figure 1a and b). This critical observation means that many human tumour sera contain antibodies to a 53 000 molecular weight protein that is not p53. If this control cell line had not been included these sera would certainly have been considered positive. That the protein present in the p53-negative cell line HS913T is not p53 or antigenically closely related to it has been confirmed by our failure to immunoprecipitate it with a whole panel of other anti-p53 antibodies, both monoclonal and polyclonal, recognising discrete epitopes on p53. This non-p53 53 000 molecular weight protein recognised by the tumour-bearing sera is also present in the p53-negative cell line SOAS 2, which intense genetic analysis has confirmed completely lacks p53 mRNA. Taking all our data together, in

**Figure 1** Immunoprecipitation from [$^{35}$S]methionine-labelled HOS cell extract (**a**) and HS913T cell extract (**b**). Immunoprecipitation with control monoclonal antibody DO-1 (lane 1 in **a** and **b**), with patient sera no. 3 (lane 2 in **a** and **b**) and with patient sera no. 12 (lane 3 in **a** and **b**).

**Table I** Detection of anti-p53 antibodies in sera from breast cancer patients

| | Human tumour cell lines | | | |
|---|---|---|---|---|
| *Methods used* | *HS913T (no p53 expression)* | *HOS (high level of mutant P53)* | *A431 (high level of mutant P53)* | *Pure p53*[a] |
| Immunoprecipitation on labelled protein | 12% | 13% | 13 | ND |
| Western blotting | 0% | 0% | 0 | 0% |
| ELISA | ND | ND | ND | 25% |

ND, not done. [a]p53 purified from insect cells as described in Materials and methods.

our sera from breast cancer patients we could with certainty identify genuine p53-specific circulating antibodies in only one patient (1%). This serum was positive in the ELISA and immunoprecipitation assay but not in the immunoblotting assays. If, however, we had only considered our ELISA results with pure p53 protein or our immunoprecipitation data without the p53 null cell line controls then we would have detected 25% and 13% 'positive cases'.

### Discussion

The p53 status of breast cancer patients has been assessed by various studies at the DNA and protein levels, and the data so far suggest that there is a correlation between high p53 levels and unfavourable disease prognosis (Thor *et al.*, 1992; Allred *et al.*, 1993; Vojtesek *et al.*, 1993). The possible immune self recognition of the aberrant p53 protein and formation of specific antibodies in patient sera could potentially provide a new approach in detecting p53 tumour-related changes by means of a relatively simple serological assay. However, while the immunogenicity of p53 protein in xenogenic systems has been well established and can be documented by a number of monoclonal antibodies developed in various animal species immunised with p53 protein, the frequency of the production of autoantibodies to p53 in humans is still unclear.

We report on the failure to detect circulating p53 autoantibodies in a clinically meaningful number of mammary cancer patients using three different immunoassays. Out of 100 breast cancer patient sera collected from two areas with substantially different demographic characteristics (i.e. 80 sera from Europe and 20 sera from South America), circulating p53 autoantibodies were clearly noted in only one patient of European origin. This result is in apparent disagreement with the findings of other authors (Crawford *et al.*, 1982; Caron de Fromentel *et al.*, 1987; Angelopoulou and Diamandis, 1992; Davidoff *et al.*, 1992; Winter *et al.*, 1992; Lubin *et al.*, 1993; Schlichtholz *et al.*, 1994), who found p53 serum antibodies in a much higher, though variable, proportion of breast cancer patients, ranging from 5% to 25% of positive cases. Undoubtedly, differences in the assay systems, the number of patients examined and genetic heterogeneity of the patients studied might account for these variations in results. Because of the limitations of individual assays and possible misinterpretation of results, we included additional negative controls, for example the use of lysates from cells not expressing p53 enabled us to exclude several false-positive findings. Our ELISA showed that 25% of the sera, regardless of whether they originated from patients with 'p53-positive' or 'p53-negative' tumours, have antibodies which apparently recognise human p53 protein. As this was unexpected, we decided to confirm these 'positives' using immunoprecipitation of labelled protein as this technique could determine whether the antibodies recognise specific conformational forms of the p53 protein. Using immunoprecipitation we could conclude that at least some of the antibodies detected in the sera of the patients are probably induced primarily against other immunogens and could cross-react or not react at all with p53 protein. In particular, we note the high frequency of sera that contain antibodies to protein of 53 000 molecular weight that is not p53.

The lack of correlation between ELISA p53 antibody-positive sera and p53 expression in the matched tumour tissue might support our speculation that either a certain proportion of antibodies detected by ELISA reflect the B-cell response to an as yet unknown antigen of approximately 53 kDa molecular weight or the specific p53 antibody formation could in some individuals represent an autoimmune phenomenon not related to the tumorigenic process. The latter assumption is supported by a study by C Vennegoor *et al.* (personal communication), who showed that sera from 4 out of 25 healthy persons (16%) contain autoantibodies to linear epitopes of wild-type p53 protein. Further studies of the presence of p53 antibodies in human sera will require analyses of healthy control sera and very stringent serological controls in order to be certain that a completely accurate distinction between positive and negative samples is made. Only then can the potential clinical significance of the finding be judged. While it is clear that some patients do produce antibodies that bind p53, our own results and those of Hassapoglidou and Diamandis (1992), in contrast to those of Crawford *et al.* (1982), Caron de Fromentel *et al.* (1987), Angelopoulou and Diamandis (1992), Davidoff *et al.* (1992), Winter *et al.* (1992), Lubin *et al.* (1993) and Schlichtholz *et al.* (1994), seem to argue that the fraction of true positives in these breast cancers is so low that the determination of p53 antibody status is unlikely to be of major use in the detection of occult disease. An important factor that could control the level of genuine anti-p53 antibodies present in sera may be the stage of the disease, since our study has specifically focused on the arguably clinically more relevant stages. To confirm this hypothesis we plan to carry out further studies of late-stage patient sera.

**Acknowledgements**

We are grateful to SM Picksley for valuable comments on the manuscript. This study was supported by Grants Nos. 1460-3 and 1459-3 from the Internal Grant Agency of the Czech Ministry of Health and by Grant No. 312/93/2329 from the Grant Agency of the Czech Republic. R Nenutil was supported by Grant No. 0861-2 from the Internal Grant Agency of the Czech Ministry of Health. DP Lane is supported by the Cancer Research Campaign.

### References

ALLRED DC, CLARK GM, ELLEDGE R, FUQUA SAW, BROWN RW, CHAMNESS GC, OSBORNE CK AND McGUIRE WL. (1993). Association of p53 protein expression with tumour cell proliferation rate and clinical outcome of node-negative breast cancer. *J. Natl Cancer Inst.*, **85**, 200-206.

ANGELOPOULOU K AND DIAMANDIS EP. (1992). Autoantibodies against the p53 tumor suppressor gene product quantified in cancer patient serum with time-resolved immunofluorometry. *Cancer*, **6**, 315-321.

CARON DE FROMENTEL C, MAY-LEVIN F, MOURIESSE H, LEMERLE J, CHANDRASEKARAN K AND MAY P. (1987). Presence of circulating antibodies against cellular protein p53 in a notable proportion of children with B-cell lymphoma. *Int. J. Cancer*, **39**, 185-189.

CRAWFORD LV, PIM DC AND BULBROOK RD. (1982). Detection of antibodies against the cellular protein p53 in sera from patients with breast cancer. *Int. J. Cancer*, **30**, 403-408.

DAVIDOFF AM, IGLEHART JD AND MARKS JR. (1992). Immune response to p53 is dependent upon p53/HSP70 complexes in breast cancers. *Proc. Natl Acad. Sci. USA*, **89**, 3439-3442.

GANNON JV, GREAVES R, IGGO R AND LANE DP. (1990). Activating mutations in p53 produce a common conformational effect. A monoclonal antibody specific for the mutant form. *EMBO J.*, **9**, 1595-1602.

HARLOW EE AND LANE DP. (1988). *Antibodies: A Laboratory Manual*. Cold Spring Harbor Laboratory Press: Cold Spring Harbor, NY.

HASSAPOGLIDOU S AND DIAMANDIS EP. (1992). Antibodies to the p53 tumour suppressor gene product quantified in cancer patient serum with a time-resolved immunofluorometric technique. *Clin. Biochem.*, **25**, 445-449.

HUPP TR, MEEK DW, MIDGLEY CA AND LANE DP. (1992). Regulation of the specific DNA binding function of p53. *Cell*, **71**, 875-886.

LUBIN R, SCHLICHTHOLZ B, BENGOUFA D, ZALCMAN G, TREDANIEL J, HIRSCH A, CARON DE FROMENTEL C, PREUDHOMME C, FENAUX P, FOURNIER G, MANGIN P, LAURENT-PUIG P, PELLETIER G, SCHLUMBERGER M, DESGRANDCHAMPS F, LE DUC A, PEYRAT JP, JANIN N, BRESSAC B AND SOUSSI T. (1993). Analysis of p53 antibodies in patients with various cancer define B-cell epitopes of human p53: distribution on primary structure and exposure on protein surface. *Cancer Res.*, **53,** 5872–5876.

MIDGLEY CA, FISHER CJ, BARTEK J, VOJTESEK B, LANE DP AND BARNES DM. (1992). Analysis of p53 expression in human tumours: an antibody raised against human p53 expressed in *Escherichia coli*. *J. Cell Sci.*, **101,** 183–189.

SCHLICHTHOLZ B, TREDANIEL J, LUBIN R, ZALCMAN G, HIRSCH A AND SOUSSI T. (1994). Analyses of p53 antibodies in sera of patients with lung carcinoma define immunodominant regions in the p53 protein. *Br. J. Cancer*, **69,** 809–816.

THOR AD, MOORE II, DH, EDGERTON SM, KAWASAKI ES, REIHSAUS E, LYNCH HT, MARCUS JN, SCHWARTZ L, CHEN LING-CHUN, MAYALL BH AND SMITH HS. (1992). Accumulation of p53 tumor suppressor gene protein: an independent marker of prognosis in breast cancers. *J. Natl Cancer Inst.*, **84,** 845–855.

VOJTESEK B, BARTEK J, MIDGLEY CA AND LANE DP. (1992). An immunochemical analysis of the human nuclear phosphoprotein p53: new monoclonal antibodies and epitope mapping using recombinant p53. *J. Immunol. Methods*, **151,** 237–244.

VOJTESEK B, FISHER CJ, BARNES DM AND LANE DP. (1993). Comparison between p53 staining in tissue sections and p53 proteins levels measured by an ELISA technique. *Br. J. Cancer*, **67,** 1254–1258.

WINTER SF, MINNA JD, JOHNSON BE, TAKAHASHI T, GAZDAR AF AND CARBONE DP. (1992). Development of antibodies against p53 in lung cancer patients appears to be dependent on the type of p53 mutation. *Cancer Res.*, **52,** 4168–4174.